# Efficacy of i-PRF in regenerative endodontics therapy for mature permanent teeth with pulp necrosis: study protocol for a multicentre randomised controlled trial

**DOI:** 10.1186/s13063-021-05401-7

**Published:** 2021-07-06

**Authors:** Yuee Liang, Rongyang Ma, Lijuan Chen, Xingzhu Dai, Shiya Zuo, Weiyi Jiang, Naiming Hu, Zilong Deng, Wanghong Zhao

**Affiliations:** Department of Stomatology, Nanfang Hospital, Southern Medical University, No. 1838, Guangzhou Avenue North, Guangzhou, 510515 China

**Keywords:** Dental pulp necrosis, Regenerative endodontics therapy, Pulp regeneration, Blood clot, Injectable platelet-rich fibrin, Mature permanent teeth

## Abstract

**Background:**

Dental pulp necrosis, a common health problem, is traditionally treated with root canal therapy; however, it fails in restoring the vitality of damaged pulp. Most studies regarding regenerative endodontic therapy (RET) are limited to the treatment of immature necrotic teeth. Given that injectable platelet-rich fibrin (i-PRF) has shown great potential in regenerative medicine as a novel platelet concentration, this study is designed to explore whether i-PRF can serve as a biological scaffold, extending the indications for RET and improving the clinical feasibility of RET in mature permanent teeth with pulp necrosis.

**Methods:**

This is a randomised, double-blind, controlled, multicentre clinical trial designed to evaluate the clinical feasibility of RET for mature permanent teeth with pulp necrosis and to compare the efficacy of i-PRF and blood clots as scaffolds in RET. A total of 346 patients will be recruited from three centres and randomised at an allocation ratio of 1:1 to receive RET with either a blood clot or i-PRF. The changes in subjective symptoms, clinical examinations, and imaging examinations will be tracked longitudinally for a period of 24 months. The primary outcome is the success rate of RET after 24 months. The secondary outcome is the change in pulp vitality measured via thermal and electric pulp tests. In addition, the incidence of adverse events such as discolouration, reinfection, and root resorption will be recorded for a safety evaluation.

**Discussion:**

This study will evaluate the clinical feasibility of RET in mature permanent teeth with pulp necrosis, providing information regarding the efficacy, benefits, and safety of RET with i-PRF. These results may contribute to changes in the treatment of pulp necrosis in mature permanent teeth and reveal the potential of i-PRF as a novel biological scaffold for RET.

**Trial registration:**

ClinicalTrials.govNCT04313010. Registered on 19 March 2020

**Supplementary Information:**

The online version contains supplementary material available at 10.1186/s13063-021-05401-7.

## Background

Dental pulp necrosis has become one of the most common health problems being the sequelae of caries, trauma, and inappropriate endodontic treatments [1, 2]. Necrosis of the dental pulp not only affects long-term tooth survival and preservation, but also can serve as a source of bacteria leading to the infection of periapical tissue and even the maxillofacial space. In addition, there is a strong correlation between oral infections and systemic diseases such as cardiovascular diseases and diabetes mellitus [3].

To date, root canal therapy (RCT) is the most acceptable treatment for pulp necrosis, based on the replacement of inflamed or necrotic pulp tissue with artificial filling materials [4]. However, this conventional therapy does not restore the vitality of damaged pulp or reconstitute the neurovascular system [5, 6]. Therefore, regenerative endodontic therapy (RET) has attracted increasing attention with the development of tissue engineering. RET applies the concept of tissue engineering (stem cells, biomimetic scaffolds, and bioactive growth factors) to achieve revascularization, innervation, and restoration of odontoblastic layers [5, 7, 8]. Unlike root canal therapy, the disinfected canals are filled with vital tissue in RET rather than biocompatible, nonvital foreign materials [9]. While several studies have explored the potential of pulp regeneration for infected teeth, most studies are limited to the treatment of immature necrotic teeth [10]. For mature permanent teeth, vital pulp is also of great importance in enhancing the immune system in root canals, promoting dentin deposition under chronic stimuli, and preventing discolouration [6]. But most of the reports of pulp regeneration in mature teeth are case reports or case series [6, 9, 11, 12] as mature teeth have fewer stem/progenitor cells, have narrower apical pathways for cell migration, and are more difficult to disinfect [4, 6, 13]. Hence, it is of great urgency to develop an improved RET procedure to maximise the regeneration potential of mature teeth and extend the indications of RET.

Scaffolds play a key role in RET, as they provide a favourable microenvironment for stem cells to migrate, proliferate, and differentiate [14–17]. Blood clot revascularization (BCR) is the most common revascularization method, in which the blood clot can serve as a scaffold and is formed by an endodontic file introduced into the root canal beyond the apical foramen to provoke bleeding from the periapical tissue [13, 18–20]. The evoked-bleeding technique stimulates surviving dental pulp stem cells near the root apex to migrate into the root canal space, such as periodontal ligament stem cells and bone marrow mesenchymal stem cells [6, 21, 22]. However, blood clots are a poor source of growth factors [19], and the precise technique required to control the speed and volume of bleeding has limited its applications in clinical practice [20]. Furthermore, several studies regarding mature necrotic teeth reported that BCR resulted in a negative response to pulp sensibility after 8–26 months [23]. Therefore, the development of a bioscaffold with high concentration of pro-regenerative factors is an important topic in the field of pulp regeneration.

Platelet products have been used in tissue regeneration and dentistry for over two decades and have shown great potential in providing an optimal scaffold for pulp regeneration [24–27]. Platelet-rich fibrin (PRF), the second-generation platelet concentrate, is a kind of purely natural autologous fibrin matrix with trapped platelets and leukocytes, guaranteeing the prolonged release of growth factors and cytokines [19, 28, 29]. However, the solid-state of PRF limits its applications [30]. There is controversy regarding the performance of PRF in RET compared to that of the conventional blood clots and the first-generation platelet concentrate, platelet-rich plasma (PRP) [17, 20]. As the low-speed centrifugation concept (LSCC) was developed, injectable platelet-rich fibrin (i-PRF) was developed as a novel liquid blood derivative in 2014. This liquid blood derivative is enriched with high concentrations of platelets, leukocytes, and growth factors [31–33]. Recent studies have reported that i-PRF provides a three-dimensional fibrin-dynamic gel embedded with platelets, leukocytes, type-I collagen, osteocalcin, and growth factors [31] with the potential to modulate inflammatory processes [34], exert anti-bacterium and anti-biofilm activities [35, 36], and promote osteogenesis [37–39]. Compared to PRF, i-PRF results in better bone regeneration [39], treatment of gingival recession defects [40], and elimination of endodontic infection [36]. However, there is no high-quality evidence regarding whether i-PRF, as an improved platelet product, could serve as an optimised scaffold in RET. At the same time, the lack of a standard protocol for RET with i-PRF also limits the use of i-PRF in RET, as different protocols may result in different treatment outcomes [5, 41, 42].

Therefore, the main aim of this randomised, double-blind, controlled, multicentre clinical trial is further evaluating the efficacy of RET in treating mature necrotic teeth, and exploring the feasibility of i-PRF as a scaffold in RET.

## Methods

### Study design and objectives

**Fig. 1:**
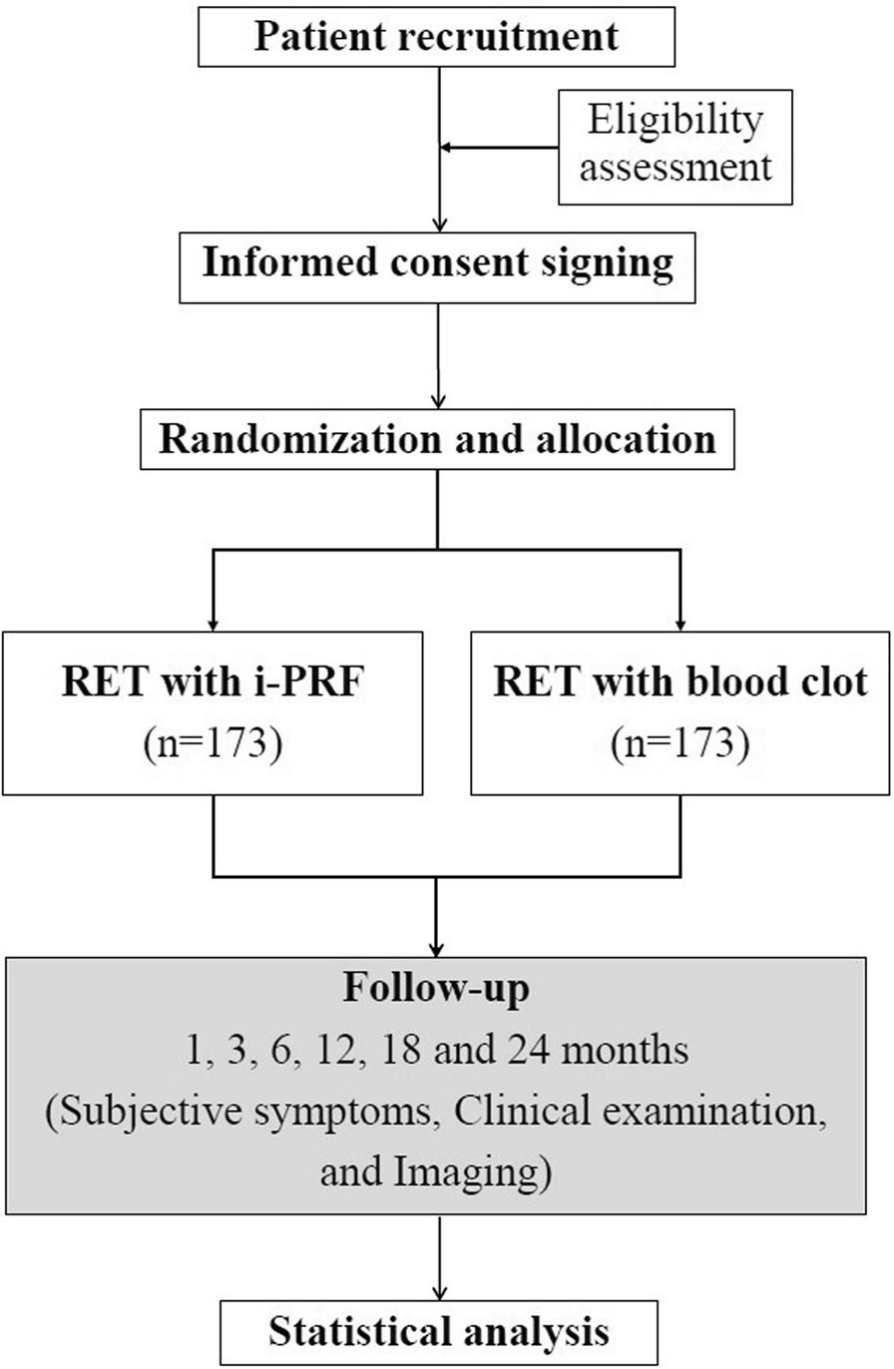
Study flowchart

**Fig. 2:**
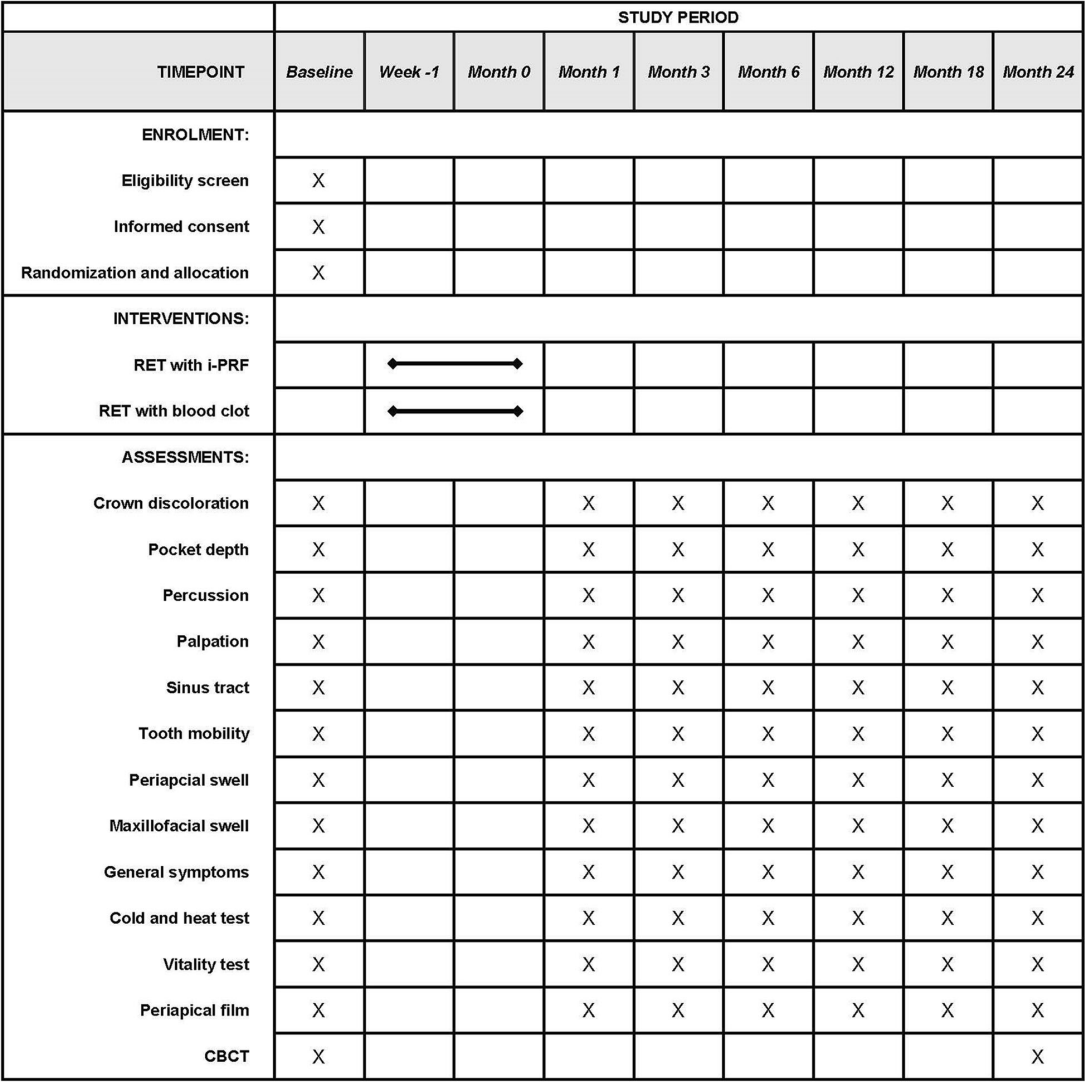
Schedule of intervention and assessments. All study-related procedures are outlined in this figure. RET, regenerative endodontic therapy; CBCT, cone beam computed tomography

This is a randomised, double-blind, parallel positive control, multicentre clinical trial (Fig. 1) that aims to compare the efficacy of i-PRF and blood clots (BCs) as scaffolds in RET for mature permanent teeth with pulp necrosis. The protocol will be reported according to the Standard Protocol Items: Recommendations for Interventional Trials (SPIRIT) statement (Additional file 1). After informed consent acquisition, all eligible patients (*n* = 346) will be randomly divided into two groups at an allocation ratio of 1:1. Patients in the test group will receive RET with i-PRF while those in the control group will receive conventional RET with BC. The follow-up period will be 24 months, and clinical data will be collected at 1, 3, 6, 12, 18, and 24 months after surgery. Figure 2 outlines the schedule of events for patients in the trial.

The primary objective of this study is to evaluate the efficacy of RET and RET with i-PRF for the treatment of mature permanent teeth with pulp necrosis. The secondary aim is to carefully investigate the factors influencing the clinical outcomes of RET in mature permanent teeth with pulp necrosis, and at the same time, to explore the standard RET protocol for treating mature permanent teeth with pulp necrosis.

### Study setting and recruitment

The study will be carried out at three centres of Southern Medical University: NanFang Hospital, Stomatological Hospital, and Shenzhen Hospital. Two dentists from each centre will participate in this trial after receiving standardised training to obtain a comprehensive overview of the rationale and strategy of the standard intervention procedure. A total of 346 patients will be recruited via outpatient hospital clinics by researchers of the above centres. After an eligibility assessment and informed consent, the patients will be randomly assigned to the control group or the experimental group at a ratio of 1:1. Patients who agree to participate in this study will sign an informed consent form.

#### Inclusion criteria

1. Agree to participate in this study and provide informed consent
2. Age 10–59 years at the time of enrolment
3. At least one mature permanent tooth diagnosed with pulp necrosis (no response to pulp vital test) with or without periapical lesions
4. Radiologic confirmation (cone beam CT (CBCT)) of a single root necrotic tooth with a single root canal

#### Exclusion criteria

1. Teeth with severe coronal defects that require post and core as final restorations
2. Non-restorable teeth
3. Teeth with root fractures or split roots
4. Presence of other pathological root resorptions
5. Presence of periodontitis
6. Allergies to any medications or materials necessary to complete the procedures
7. Presence of dental dysplasia or other oral genetic disorders
8. Women who are pregnant or lactating, or women who plan to become pregnant in the subsequent 2 years
9. Presence of dental phobia
10. Presence of mental disorders
11. A history of systemic diseases that may alter immune function
12. Medical conditions and/or receiving medications that affect healing or blood coagulation
13. Participation in other clinical studies within the past 3 months
14. Patients who are deemed unsuitable to participate in the study by investigators

#### Dropout criteria

1. Presence of serious adverse events that doctors believe should lead to termination of trial participation, such as severe internal or external root resorption and tooth fracture
2. Poor clinical compliance
3. Withdrawal of consent for study participation by the patient

### Randomisation, allocation, and blinding

Eligible patients will be randomly assigned to one of two groups at an allocation ratio of 1:1. The randomisation sequence and grouping will be generated and stored by one researcher who will not be involved directly in participant screening, enrolment, or assessment, using SPSS 20.0 software (IBM Co., Armonk, NY, USA). All random numbers will be listed sequentially, and a set of 10 numbers will be arranged as a cluster (the first 5 for the test group and the last 5 for the control group). Then, random numbers and grouping are inserted in opaque and sealed envelopes sequentially. After all the envelopes have been sealed, they will be distributed to each research centre. Only dentists can open the envelope to check the group allocation and perform the interventions according to the instructions of this study. As a double-blind trial, the patients and outcome evaluators will be blinded to the group assignment until the completion of the study. As dentists cannot be blinded to treatment allocation due to the notable differences in the treatment methods, they will not be allowed to discuss the type of intervention with either patients or outcome evaluators.

### Interventions

According to the latest RET operating guidelines issued by the American Academy of Endodontics (AAE) [43] and the European Society of Endodontics (ESE) [44], the standardised operating procedure of RET requires two treatment visits. During the first appointment, infection is controlled and inflammation is relieved. Pulp regeneration and revascularization is accomplished during the second appointment. All RET procedures will be performed under a dental microscope, except anaesthesia and rubber dam placement steps.

#### First Appointment

With the aim of infection control and inflammation relief, the first appointment will be the same for all patients in this study. The first appointment will include the following components:

1. *Anaesthesia and isolation*. After local anaesthesia with articaine hydrochloride and epinephrine tartrate injection (with 1:100,000 adrenaline [Produits Dentaires Pierre Rolland, Merignac, France]), the tooth is isolated with a rubber dam and disinfected.
2. *Access preparation*. All decayed areas of the tooth will be removed, and the pulp chamber will be completely unroofed.
3. *Root canal preparation*. The working length and the initial apical file will be determined.
4. *Root canal irrigation*. As canal disinfection relies considerably on chemical irrigants, the needle will be placed with the side-port vents into the apical third of the tooth and irrigation will be accomplished using needles with a slow infusion rate. The canal will be copiously irrigated in three steps using ultrasonic irrigation with interspersed drying with sterile paper points. A 5-min irrigation with 20 mL 1.0% sodium hypochlorite (NaClO) (Longly biomedical, Wuhan, China) will be followed by a simple irrigation with 5-mL sterile saline solution (Baxter Healthcare, Shanghai, China), ending with a 5-min irrigation with 20 mL 17% ethylene diamine tetraacetie acid (EDTA) (Longly biomedical, Wuhan, China).
5. *Medicated intra-pulpal dressings*. To restrict bacterial regrowth and supply continued disinfection, the root canal will be filled homogeneously to the working length with calcium hydroxide paste (CALXYL X-Ray Visible, OCO Praparate GmBh, Dirmstein/Pfalz, Germany) [45]. Then, glass ionomer (GC Fuji IX, GC Corporation, Tokyo, Japan) will be placed onto the intracanal dressing.

#### Second Appointment

**Fig. 3:**
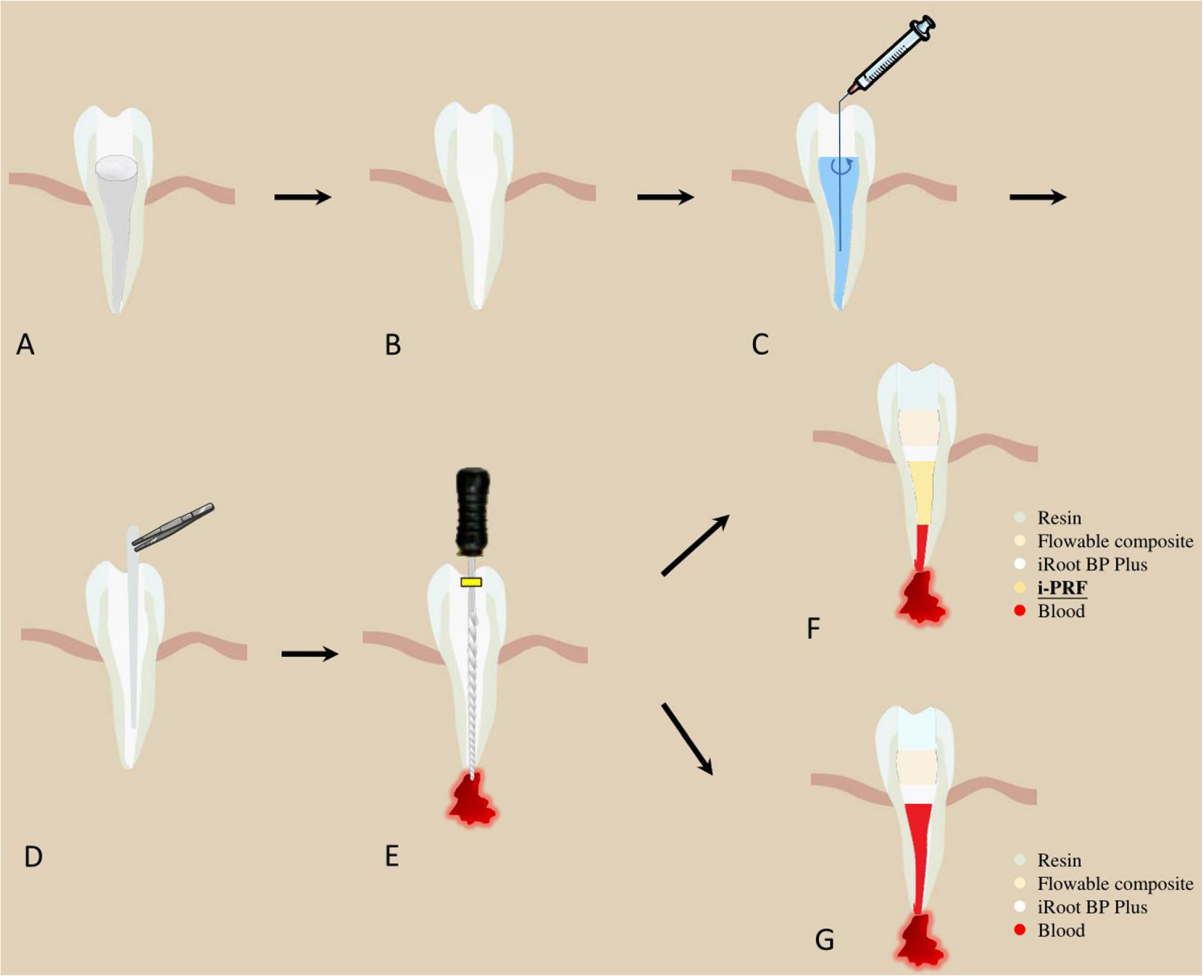
Regenerative endodontics therapy procedures. The steps of regenerative endodontics therapy (RET) with injectable platelet-rich fibrin (i-PRF) and with blood clot are shown. **A** After 2–4 weeks of intercanal medication, **B** the temporary seal is removed. **C** The root canal is irrigated and **D** dried with sterile paper points. **E** Bleeding is provoked. **F** i-PRF and cavity restoration are performed in the test group. **G** Blood clot and cavity restoration are performed in the control group

After 2–4 weeks, patients will return for a second visit. If the symptoms are not relieved, the root canals will be re-treated with calcium hydroxide. Patients with no symptoms will proceed to the next RET treatment steps (Fig. 3).

**Fig. 4:**
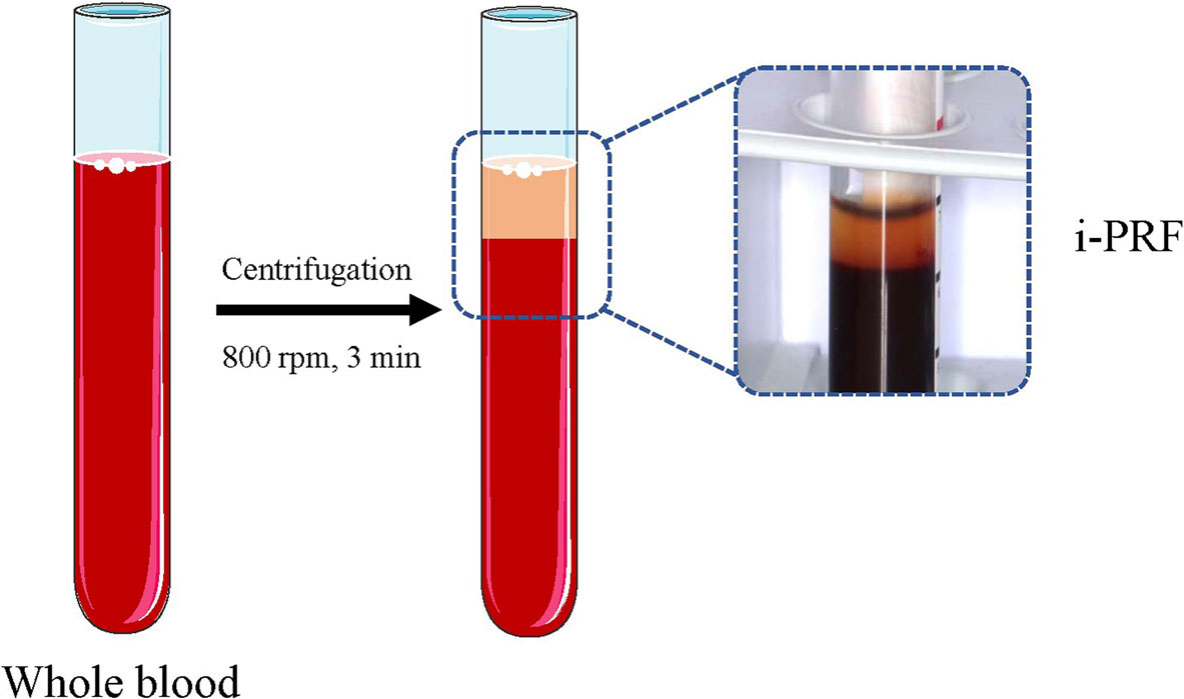
The preparation of injectable platelet-rich fibrin. After centrifugation (800 rpm, 3 min), injectable platelet-rich fibrin (i-PRF) is collected from the upper yellow fluid liquid

1. Cleaning, anaesthesia, field isolation, disinfection of the operating field, and temporary seal removal
2. *Root canal irrigation*. The canal will be irrigated with 20 mL of 17% EDTA for 5 min to flush out the calcium hydroxide paste, followed by irrigation with sterile saline solution to decrease the cytotoxicity of EDTA. After drying the canal using sterile paper points, the two groups of patients will experience different revascularization procedures.
3. *PRF preparation*. To prepare PRF, 10 mL of whole blood from the median cubital vein will be drawn into 10-ml plastic tubes (Kangjian Medical, Jiangsu, China) without anticoagulant reagent and centrifuged at 800 rpm for 3 min with Centrifuge (Process for PRF, FRANC). After centrifugation, the upper yellow fluid liquid (i-PRF) will be collected as close as possible to the layer of red cells (Fig. 4).
4. *RET with i-PRF (the test group)*. K-files will be intentionally used to violate the periapical tissues via overinstrumentation up to 2–3 mm beyond the apical foramen to provoke bleeding. However, only the apex third of the root canal will be filled with blood. The i-PRF will be injected into the root canal to a level 3 mm below the cementoenamel junction (CEJ). After a 10–15-min period for coagulation, the PRF will be covered by a 3-mm layer of iRoot BP Plus (Innovative Bioceramix Inc., Vancouver, Canada).
5. *RET with BC (the control group)*. K-files will be intentionally used to violate the periapical tissues via overinstrumentation up to 2–3 mm beyond the apical foramen to provoke bleeding. The blood will be permitted to fill the canal space to a level 3 mm below the CEJ. After a 10–15-min period for coagulation, the BC will be covered by a 3-mm layer of iRoot BP Plus.
6. *Cavity restoration*. A layer of self-adhering flowable composite (Kerr Dyad Flow, Kerr, CA, USA) will be applied on top of the iRoot BP Plus layer as a cavity liner, and the final filling will be accomplished with resin with adhesive for permanent restoration.

### Outcome measures

Variables will be measured at baseline and at 1, 3, 6, 12, 18, and 24 months after the treatment. At each appointment, a clinical examination, a periapical radiograph, and pulp tests (thermal and electric) will be performed. CBCT images will be obtained at baseline and 24 months after the treatment to evaluate the apical shadow. The primary outcome of this study is the success rate of RET 24 months postoperatively, which will be obtained by evaluating the clinical and radiographic outcomes. The secondary outcomes include the subjective response from the patient to thermal and electric stimuli, which reflects the responsiveness of regenerative pulpal sensory neurons. Safety will be evaluated via the analysis of the presence of adverse events during the study. The evaluators will complete standardised training prior to the study period. Two trained dentists will independently evaluate and disagreements will be resolved through a discussion with a third evaluator.

#### Primary outcome measures

The therapeutic effect evaluation criteria are the following:

1. *Success*. After RET, the tooth is asymptomatic, with an absence of percussion pain, bone adhesion percussion sound, loosening, tenderness, mucosal swelling, sinus, or fistula. The radiographic examination demonstrates the absence of an apical shadow or a reduced apical shadow.
2. *Failure*. After RET, the tooth is symptomatic, with symptoms of apical infection such as pain and fistula. The radiographic examination demonstrates a new-onset apical shadow or an enlarged apical shadow.

#### Secondary outcome measures

The preoperative results of the thermal pulp test and the electric pulp test will be recorded to ensure pulp necrosis of the involved tooth. The test results during the follow-up will be used to determine if the teeth included in the study regain sensitivity.

1. The results of thermal pulp test are as follows:
  - *Normal*. The test tooth is considered normal if the patient responds similarly to when a control tooth is tested.
  - *Sensitive*. The test tooth is considered sensitive if the patient experiences more pain than when a control tooth is tested.
  - *Dull*. The test tooth is considered dull if the patient has a much slower, reduced response when stimulated compared to when a control tooth is tested at the same temperature.
  - *No response*. The tested tooth is considered to have no response if the patient does not respond to stimulation.
2. The results of electric pulp test are as follows:
  - The numerical reading is recorded when the patient elicits a response. To ensure consistency, each tooth will be tested twice and the average result will be used in the analyses.

#### Safety evaluation

All information regarding adverse events during the study will be recorded in detail, including symptoms, signs, onset time, and severity. Some possible adverse events that may be attributed to RET include reinfection of the root canal, external root resorption, internal root resorption, and discolouration.

### Sample size and statistical methods

#### Sample size

The primary outcome measure is the success rate at 24 months after treatment. According to nQuery Advenced 8.6 software (Statistical Solutions Ltd., Cork, Ireland), a sample size of 346 (173 for each group) patients is required when α = 0.05 and the test power is 80%.

#### Data collection methods and data management

Data associated with this study will be collected in the standardised case report form (CRF) for the outcome analysis, and a specific supervisor will be responsible for reviewing the integrity, accuracy, and consistency of the data. To ensure the accuracy of data entry, two researchers will be responsible for entering the data independently and data query forms (DQF) will be resolved by tracing the source data. All the data will be registered in document clouds, and only authorised researchers will have access. Data monitoring committee consists of the Department of Biomedical Statistics, Southern Medical University, independent from the sponsor and competing interests.

As for the audition of this study, the frequency is once a year. During the audition, the project organisation will review the test process and make a comprehensive evaluation. The process will be independent of investigators and the sponsor.

#### Statistical analysis

The data will be analysed by an independent statistician using SPSS 20.0 software (IBM Co., Armonk, NY, USA) at the end of the study to evaluate the evolution. The continuous variables will be described as the mean (standard deviation) or median and analysed using parametric methods. Categorical variables will be described as percentages and analysed using non-parametric methods. The level of significance will be *P* < 0.05, and 95% confidence intervals will be calculated.

1. *Baseline analysis*

The two groups will be comparable in terms of sex, age, tooth position, disease type, and apical hole size. Quantitative variables will be analysed using a one-way analysis of variance (independent sample t-test) or non-parametric methods if the data do not meet and cannot be transformed to meet parametric assumptions. Qualitative variables will be analysed using the Wilcoxon rank-sum test. A principal component analysis will be performed using multinomial logistic regression to adjust for potential confounders.

(2) *Main outcome analysis*

The inter-group comparison of success rates will be analysed using the chi-square test. If there are baseline variables influencing the comparability of the two groups, a logistic regression model will be used to eliminate the effects of these confounders on the success rate and the odds ratio (OR) will be calculated. For the missing data, the last observation carry forward method will be adopted to fill the validity analysis, that is, the case data that fails to observe the whole treatment process will be transferred to the final result of the test with the last observation data.

(3) *Secondary outcome analysis*

For intra-group comparisons, McNemar’s test will be used to analyse the clinical outcomes of different centres. For inter-group comparisons, the secondary outcome will be analysed using the Chi-square test.

(4) *Safety evaluation analysis*

The adverse events that occur during the study period will be listed for each group and compared using the chi-square test. The security evaluation does not evaluate missing data.

### Ethics and dissemination

The protocol has been approved by the Medical Ethics Committee of NanFang Hospital of Southern Medical University (reference NFEC-2019-233) and registered at ClinicalTrials.gov (reference NCT04313010). Any amendments to the protocol will be reviewed and approved by the ethics committee and funding support departments. Written informed consent will be obtained from all patients. The results will be published in the peer-reviewed journal and presented at conference presentations.

The Data Management Coordinating Centre will oversee the intra-study data sharing process, with the involvement of the Data Management Subcommittee. Only the principal investigator will be given access to all the data with a special password. Other project investigators will have direct access to their own site’s data sets, and other sites’ data by request. To ensure confidentiality, data dispersed to project team members will be blinded to any identifying participant information.

As for ancillary and post-trial care, all the patients who participated in the study could receive compensation from the study unit, including additional medical care, compensation or damages.

## Discussion

As decades of evidence demonstrates the benefits of RET in immature teeth, the technique has recently been extended to mature teeth as an alternative to conventional endodontic treatments [23, 46]. As RET in mature teeth is challenging, there is a lack of long-term clinical trials evaluating the feasibility of RET in mature necrotic teeth. In our study, the feasibility of RET for mature teeth can be tracked longitudinally by comprehensively considering subjective symptoms, clinical examinations, and imaging studies. The feasibility of using i-PRF, a novel and advanced PRF, as a biological scaffold for pulp regeneration will also be investigated. Therefore, this study will explore the feasibility of RET and of RET with i-PRF in mature necrotic teeth and develop a scientific treatment protocol to maximise the potential of i-PRF in RET.

Platelet products have shown great potential in regenerative medicine due to their potency in storing and releasing biologically active substances, regulating the innate immune response, and combatting infection [24–27]. Therefore, several studies regarding the potential of platelet products for pulp regeneration have been conducted. Platelet-rich plasma (PRP), 95% of which is platelets, is the first-generation platelet concentrate [47]. PRP can promote endothelial progenitor cells to form vessel-like structures [48] and the regeneration of pulp-like tissue [48, 49] in immature teeth. However, the protocol for harvesting PRP involves the use of anticoagulants and multiple centrifugation steps with high g-forces [27, 28], and growth factors are released from PRP in a burst-like kinetic manner [50, 51]. As a result, PRF, the second-generation platelet concentrate derived from patient venous blood through single-step centrifugation, has emerged as a purely natural autologous fibrin matrix with trapped platelets and leukocytes, guaranteeing the prolonged release of growth factors and cytokines [19, 28, 29]. Since then, there are increasing researches devoted to optimising the protocol to obtain better platelet concentrates to meet different clinical needs. Leukocyte- and platelet-rich-fibrin (L-PRF), the first blood-derived PRF matrix without anticoagulants, has the potential to improve tissue healing in clinical cases [26, 31, 52–54]. Then, based on lower centrifugation speeds, advanced PRFs (A-PRF and A-PRF+) have been developed with a porous structure and more neutrophilic granulocytes [28, 29, 45]. Thus, the LSCC has been developed [55–57] to obtain PRF with a higher pro-regenerative potency. In 2014, i-PRF was developed. As a flexible matrix, i-PRF overcomes the limitations of the solid PRF matrix [31–33]. Thus, i-PRF has potential as an optimal scaffolding material in RET in mature teeth. Randomised controlled clinical trials are needed to provide high-quality evidence to evaluate the feasibility of RET with i-PRF and, at the same time, explore the standard clinical protocol.

One of the challenges of performing RET in mature permanent teeth is that there is no agreement in the size of the apical foramen for new tissue to grow into the canals. Earlier, it was been found that foramens smaller than 1 mm (measured on radiographs) would prevent the revascularization of the pulp tissue [58]. As a result, many endodontists preferred to mechanically prepare the root canal to a large apical size when performing RET on mature teeth. An animal model study reported that an apical foramen 0.32 mm in diameter did not prevent revascularization and ingrowth of new tissue into canals after transplantation [59]. A range of apical foramen diameters have been attempted in the previous reports of RET on mature teeth, including 1.0 mm [11], 0.6 mm [6], and 0.4 mm [9]. With the deepening of research, it has been revealed that enlargement of the apical foramen to at least 1 mm in diameter is unnecessary for RET [9]. Thus, in this study, we plan to use the conventional canal preparation protocol and avoid excessive preparation, which can increase the risk of root fracture.

The clinical efficacy of RET for mature necrotic teeth depends heavily on disinfection regimens. In this study, 20 mL of 1.0% NaClO will be used for irrigation with a side-vented needle and an ultrasonic irrigation device to minimise the possibility of extrusion of irrigates into the periapical space and to enhance the disinfection efficacy of NaClO. Following the recommendation from the AAE, NaClO should be flushed away from the root canal with saline to reduce any lingering toxicity [4, 60]. EDTA irrigation will also be applied in this study, as EDTA has been demonstrated to promote the survival of stem cells [61], stimulate the release of growth factors embedded in the dentin matrix, and enhance the odontogenic differentiation of migrated cells and angiogenesis [6]. As for the intercanal medication of RET, the AAE and ESE have issued differing recommendations regarding intercanal medication during RET. The AAE recommends triple antibiotic paste (TAP), consisting of metronidazole, ciprofloxacin, and minocycline, while the ESE recommends the use of non-staining calcium hydroxide due to the risk of crown discolouration caused by TAP [45, 62]. TAP cannot be fully removed from root canal systems via current irrigation techniques, potentially due to its penetration and binding to dentin [63]. Thus, to restrict bacterial regrowth and supply continued disinfection, the root canals will be filled homogeneously to the working length with calcium hydroxide in this study. In addition, unlike most existing studies, iRoot BP Plus will be used as the pulp capping material instead of mineral trioxide aggregrate (MTA), due to its satisfactory biocompatibility, sealing ability, antibacterial activity, and ability to upregulate the expression of mineralization-related genes [64].

Finally, to the best of our knowledge, this is the first multicentre clinical trial to evaluate the clinical efficacy of RET in mature necrotic teeth and to evaluate i-PRF as a novel biological scaffold for pulp regeneration. Although there is a concern that this study does not include histological or morphological analyses to determine the nature of the tissues formed in response to the treatment, this study will provide high-quality evidence guiding the future of RET practice on mature permanent teeth via the comprehensive evaluation of symptoms, clinical evaluations, and radiographic assessments. Therefore, our study will contribute to explore the maximum potential of RET and RET with i-PRF in mature necrotic teeth.

## Trial status

This trial is in the process of recruiting participants.

## Supplementary Information

**Additional file 1.** SPIRIT 2013 Checklist: Recommended items to address in a clinical trial protocol and related documents.

## Data Availability

The datasets analysed during the current study are available from the corresponding author on reasonable request.

## Abbreviations

RET: Regenerative endodontic therapy
i-PRF: Injectable platelet-rich fibrin
RCT: Root canal therapy
BCR: Blood clot revascularization
PRF: Platelet-rich fibrin
PRP: Platelet-rich plasma
LSCC: Low-speed centrifugation concept
BCs: Blood clots
AAE: American Academy of Endodontics
ESE: European Society of Endodontics
CEJ: Cementoenamel junction
CBCT: Cone beam computed tomography
NaClO: Sodium hypochlorite
EDTA: Ethylene diamine tetraacetie acid
CRF: Case report form
DQF: Data query forms
OR: Odds ratio
TAP: Triple antibiotic paste
MTA: Mineral trioxide aggregrate
