# Supplementary material for: Efficacy of i-PRF in regenerative endodontics therapy for mature permanent teeth with pulp necrosis: study protocol for a multicentre randomised controlled trial
Source: Trials. 2021 Jul 6;22:436. doi: 10.1186/s13063-021-05401-7 (PMC8261915; doi:10.1186/s13063-021-05401-7)
Supplement: Supplementary file 1 — Additional file 1. SPIRIT 2013 Checklist: Recommended items to address in a clinical trial protocol and related documents. [file 13063_2021_5401_MOESM1_ESM.doc]

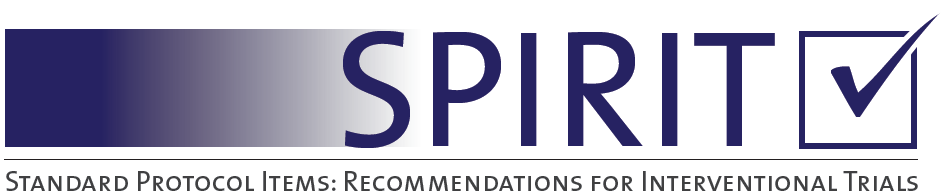


SPIRIT 2013 Checklist: Recommended items to address in a clinical trial protocol and related documents*

| Section/item | Item No | Description | Addressed on page number |
| --- | --- | --- | --- |
| **Administrative information** | | |  |
| Title | 1 | Descriptive title identifying the study design, population, interventions, and, if applicable, trial acronym Efficacy of i-PRF in regenerative endodontics therapy for mature necrotic teeth: study protocol for a multicentre randomised controlled trial | __Page 1__ __ |
| Trial registration | 2a | Trial identifier and registry name. If not yet registered, name of intended registry Protocol ID: NFEC-2019-233; ClinicalTrials.gov ID: NCT04313010; Title: Effectiveness of Regenerative Endodontics Therapy for Single-rooted Mature Permanent Tooth With Pulp Necrosis | _ Page 2_ __ _ |
|  | 2b | All items from the World Health Organization Trial Registration Data Set All WHO Trial Registration Data requirements are met with the trial’s registration in the ClinicalTrials.gov. Trial registration information is found on Page 2. | __ Page 2___ _ |
| Protocol version | 3 | Date and version identifier Date: 2021-05-04; Author: Wanghong Zhao and Zilong Deng | __Page 2__ _ |
| Funding | 4 | Sources and types of financial, material, and other support The work is funded by Clinical Research Program of Southern Medical University (No. LC2019ZD023), Clinical Research Program of Nanfang hospital, Southern Medical University (No. 2020CR029) and the President Foundation of Nanfang Hospital, Southern Medical University (No. 2019Z019). | _ Page 17 _ |
| Roles and responsibilities | 5a | Names, affiliations, and roles of protocol contributors YEL participated in the design of the study, and recruited participants. RYM drafted the manuscript. LJC recruited participants, and performed the statistical analysis. XZD and SYZ collected case data. WYJ and RYM participated in the design of outcome measures, and performed the evaluation of the clinical and radiographic outcomes. NMH assisted with participant recruitment and screening. WHZ and ZLD conceived the study, participated in its design and coordination, and helped draft the manuscript. All authors read and approved the final manuscript. | __ Page 17____ |
|  | 5b | Name and contact information for the trial sponsor The work is funded by Clinical Research Program of Southern Medical University (No. LC2019ZD023), Clinical Research Program of Nanfang hospital, Southern Medical University (No. 2020CR029) and the President Foundation of Nanfang Hospital, Southern Medical University (No. 2019Z019). | __ Page 17____ |
|  | 5c | Role of study sponsor and funders, if any, in study design; collection, management, analysis, and interpretation of data; writing of the report; and the decision to submit the report for publication, including whether they will have ultimate authority over any of these activities The study funder plays no role in study design, collection, management, analysis, or interpretation of the data, or writing/submitting the manuscript. | __ Page 17__ _ |
|  | 5d | Composition, roles, and responsibilities of the coordinating centre, steering committee, endpoint adjudication committee, data management team, and other individuals or groups overseeing the trial, if applicable (see Item 21a for data monitoring committee) Data monitoring committee consists of the Department of Biomedical Statistics, Southern Medical University. | ___ Page 12___ |
| Introduction |  |  |  |
| Background and rationale | 6a | Description of research question and justification for undertaking the trial, including summary of relevant studies (published and unpublished) examining benefits and harms for each intervention Dental pulp necrosis, a common health problem, is traditionally treated with root canal therapy; however, but it fails in restoring the vitality of damaged pulp. Most studies regarding regenerative endodontic therapy (RET) are limited to the treatment of immature necrotic teeth. Given that injectable platelet-rich fibrin (i-PRF) has shown great potential in regenerative medicine as a novel platelet concentration, this study is designed to explore whether i-PRF can serve as a biological scaffold, extending the indications for RET and improving the clinical feasibility of RET in mature permanent teeth with pulp necrosis. | ___ Page 3-4____ |
|  | 6b | Explanation for choice of comparators Scaffolds play a key role in RET, as they provide a favourable microenvironment for stem cells to migrate, proliferate, and differentiate. Scaffolds play a key role in RET, as they provide a favourable microenvironment for stem cells to migrate, proliferate, and differentiate. Blood clot revascularization (BCR) is the most common revascularization method, but blood clots are a poor source of growth factors, and the precise technique required to control the speed and volume of bleeding has limited its applications in clinical practice. And the efficacy of BCR on mature teeth is still debatable, while injectable platelet-rich fibrin (i-PRF) has shown a great potential in tissue regeneration. Therefore, RET with blood clot and RET with i-PRF were designed in this study to observe the treatment effect. | ___ Page 3-4____ |
| Objectives | 7 | Specific objectives or hypotheses Specific objectives: to further evaluate the efficacy of RET in treating mature necrotic teeth and explore the feasibility of i-PRF as a scaffold in RET; Hypotheses: the efficacy of RET with i-PRF is better than that of RET with blood clot in treating mature permanent teeth with pulp necrosis. | ___ Page 4_ __ |
| Trial design | 8 | Description of trial design including type of trial (eg, parallel group, crossover, factorial, single group), allocation ratio, and framework (eg, superiority, equivalence, noninferiority, exploratory) This trial is a randomised, double-blind, controlled, multicentre clinical trial. | Page 5, Figure 1 |
| Methods: Participants, interventions, and outcomes | | |  |
| Study setting | 9 | Description of study settings (eg, community clinic, academic hospital) and list of countries where data will be collected. Reference to where list of study sites can be obtained The study will be carried out at three centres of Southern Medical University: NanFang Hospital, Stomatological Hospital, and Shenzhen Hospital. | ___ Page 5 __ _ |
| Eligibility criteria | 10 | Inclusion and exclusion criteria for participants. If applicable, eligibility criteria for study centres and individuals who will perform the interventions (eg, surgeons, psychotherapists)  Inclusion criteria:  (1)Agree to participate in this study and provide informed consent.  (2)Age 10-59 years at the time of enrolment.  (3)At least one mature permanent tooth diagnosed with pulp necrosis (no response to pulp vital test) with or without periapical lesions.  (4)Radiologic confirmation (cone beam CT (CBCT)) of a single-root necrotic tooth with a single root canal  Exclusion criteria:  (1)Teeth with severe coronal defects that require post and core as final restorations.  (2)Non-restorable teeth.  (3)Teeth with root fractures or split roots.  (4)Presence of other pathological root resorptions.  (5)Presence of periodontitis.  (6)Allergies to any medications or materials necessary to complete the procedures.  (7)Presence of dental dysplasia or other oral genetic disorders.  (8)Women who are pregnant or lactating, or women who plan to become pregnant in the subsequent two years.  (9)Presence of dental phobia.  (10)Presence of mental disorders.  (11)A history of systemic diseases that may alter immune function.  (12)Medical conditions and/or receiving medications that affect healing or blood coagulation.  (13)Participation in other clinical studies within the past 3 months.  (14)Patients who are deemed unsuitable to participate in the study by investigators. | ____ Page 5-6 __ |
| Interventions | 11a | Interventions for each group with sufficient detail to allow replication, including how and when they will be administered According to the latest RET operating guidelines issued by the American Academy of Endodontics (AAE) and the European Society of Endodontics (ESE), the standardised operating procedure of RET requires two treatment visits. During the first appointment, infection is controlled and inflammation is relieved. Pulp regeneration and revascularization is accomplished during the second appointment. All RET procedures will be performed under a dental microscope, except anaesthesia and rubber dam placement steps. | ____ Page 7 __ |
|  | 11b | Criteria for discontinuing or modifying allocated interventions for a given trial participant (eg, drug dose change in response to harms, participant request, or improving/worsening disease)  Dropout criteria:  (1)Presence of serious adverse events that doctors believe should lead to termination of trial participation, such as severe internal or external root resorption and tooth fracture.  (2)Poor clinical compliance.  (3)Withdrawal of consent for study participation by the patient. | ____ Page 6-7_ _ |
|  | 11c | Strategies to improve adherence to intervention protocols, and any procedures for monitoring adherence (eg, drug tablet return, laboratory tests) Strategies to improve adherence to intervention protocols are not stipulated as this is a brief, two-visit intervention. And during the follow-up period, researcher will make phone calls to remind patients of the follow-up examines. | _____________ |
|  | 11d | Relevant concomitant care and interventions that are permitted or prohibited during the trial Physicians are prohibited from failing to follow a random list of interventions. | ____ __ |
| Outcomes | 12 | Primary, secondary, and other outcomes, including the specific measurement variable (eg, systolic blood pressure), analysis metric (eg, change from baseline, final value, time to event), method of aggregation (eg, median, proportion), and time point for each outcome. Explanation of the clinical relevance of chosen efficacy and harm outcomes is strongly recommended The primary outcome of this study is the success rate of RET 24 months postoperatively, which will be obtained by evaluating the clinical and radiographic outcomes. The secondary outcomes include the subjective response from the patient to thermal and electric stimuli, which reflects the responsiveness of regenerative pulpal sensory neurons. Safety will be evaluated via the analysis of the presence of adverse events during the study. | ___ Page 10 __ |
| Participant timeline | 13 | Time schedule of enrolment, interventions (including any run-ins and washouts), assessments, and visits for participants. A schematic diagram is highly recommended (see Figure) Variables will be measured at baseline and at 1, 3, 6, 12, 18, and 24 months after the treatment. At each appointment, a clinical examination, a periapical radiograph, and pulp tests (thermal and electric) will be performed. CBCT images will be obtained at baseline and 24 months after the treatment to evaluate the apical shadow. | ___ Page 7-11, Figure 2_ __ |
| Sample size | 14 | Estimated number of participants needed to achieve study objectives and how it was determined, including clinical and statistical assumptions supporting any sample size calculations In this study, the sample size of is 346 (173 for each group) patients when α = 0.05 and the test power is 80%. | ___ Page 11____ |
| Recruitment | 15 | Strategies for achieving adequate participant enrolment to reach target sample size  Each clinical centre involved in the study was chosen based on documentation for patient availability. It is, however, worthy to note the specific plans of each centre.  (1) Recruiting via outpatient hospital clinics;  (2) Advertisements;  (3) Give each participant an appropriate road subsidy and free oral examination. | ___ Page 5___ |
| **Methods: Assignment of interventions (for controlled trials)** | | |  |
| Allocation: |  |  |  |
| Sequence generation | 16a | Method of generating the allocation sequence (eg, computer-generated random numbers), and list of any factors for stratification. To reduce predictability of a random sequence, details of any planned restriction (eg, blocking) should be provided in a separate document that is unavailable to those who enrol participants or assign interventions A total of 346 patients will be recruited. After an eligibility assessment and informed consent, the patients will be randomly assigned to the control group or the experimental group at a ratio of 1:1. The randomisation sequence and grouping will be generated and stored by one researcher who will not be involved directly in participant screening, enrolment, or assessment, using SPSS 20.0 software (IBM Co., Armonk, NY, USA). All random numbers will be listed sequentially, and a set of 10 numbers will be arranged as a cluster (the first 5 for the test group and the last 5 for the control group). | ___ Page 7____ |
| Allocation concealment mechanism | 16b | Mechanism of implementing the allocation sequence (eg, central telephone; sequentially numbered, opaque, sealed envelopes), describing any steps to conceal the sequence until interventions are assigned The randomisation sequence and grouping will be generated and stored by one researcher who will not be involved directly in participant screening, enrolment, or assessment, using SPSS 20.0 software (IBM Co., Armonk, NY, USA). Then, random numbers and grouping are inserted in opaque and sealed envelopes sequentially. After all the envelopes have been sealed, they will be distributed to each research cen. Only dentists can open the envelope to check the group allocation and perform the interventions according to the instructions of this study. | ____ Page 7___ |
| Implementation | 16c | Who will generate the allocation sequence, who will enrol participants, and who will assign participants to interventions The randomisation sequence and grouping will be generated and stored by one researcher who will not be involved directly in participant screening, enrolment, or assessment, using SPSS 20.0 software (IBM Co., Armonk, NY, USA). And, participant enrolment will be performed by via outpatient hospital clinics by researchers of each centres. During the study, Only dentists can open the envelope to check the group allocation and perform the interventions according to the instructions of this study. | ____ Page 5, 7___ |
| Blinding (masking) | 17a | Who will be blinded after assignment to interventions (eg, trial participants, care providers, outcome assessors, data analysts), and how As a double-blind trial, the patients and outcome evaluators will be blinded to the group assignment until the completion of the study. | ____ Page 7___ |
|  | 17b | If blinded, circumstances under which unblinding is permissible, and procedure for revealing a participant’s allocated intervention during the trial As dentists cannot be blinded to treatment allocation due to the notable differences in the treatment methods, they will not be allowed to discuss the type of intervention with either patients or outcome evaluators. | ____ Page 7____ |
| **Methods: Data collection, management, and analysis** | | |  |
| Data collection methods | 18a | Plans for assessment and collection of outcome, baseline, and other trial data, including any related processes to promote data quality (eg, duplicate measurements, training of assessors) and a description of study instruments (eg, questionnaires, laboratory tests) along with their reliability and validity, if known. Reference to where data collection forms can be found, if not in the protocol Data associated with this study will be collected in the standardised case report form (CRF) for the outcome analysis, and a specific supervisor will be responsible for reviewing the integrity, accuracy, and consistency of the data. The data will be analysed by an independent statistician. To ensure the accuracy of data entry, two researchers will be responsible for entering the data independently and data query forms (DQF) will be resolved by tracing the source data. | _ Page 12 _ |
|  | 18b | Plans to promote participant retention and complete follow-up, including list of any outcome data to be collected for participants who discontinue or deviate from intervention protocols Data associated with this study will be collected in the standardised case report form (CRF) for the outcome analysis, and a specific supervisor will be responsible for reviewing the integrity, accuracy, and consistency of the data. | ____ Page 12___ |
| Data management | 19 | Plans for data entry, coding, security, and storage, including any related processes to promote data quality (eg, double data entry; range checks for data values). Reference to where details of data management procedures can be found, if not in the protocol Double data entry. And all the data will be registered in a documents clouds, and only authorized researchers will have access. Data monitoring committee consists of the Department of Biomedical Statistics, Southern Medical University, independent from the sponsor and competing interests. | ___ Page 12____ |
| Statistical methods | 20a | Statistical methods for analysing primary and secondary outcomes. Reference to where other details of the statistical analysis plan can be found, if not in the protocol The continuous variables will be described as the mean (standard deviation) or median, and analysed using parametric methods. Categorical variables will be described as percentages and analysed using non-parametric methods. The level of significance will be P < 0.05, and 95% confidence intervals will be calculated. | _ _ Page 12 __ |
|  | 20b | Methods for any additional analyses (eg, subgroup and adjusted analyses) The inter-group comparison of success rates will be analysed using the chi-square test. If there are baseline variables influencing the comparability of the two groups, a logistic regression model will be used to eliminate the effects of these confounders on the success rate and the odds ratio (OR) will be calculated. | __ Page 13 ___ |
|  | 20c | Definition of analysis population relating to protocol non-adherence (eg, as randomised analysis), and any statistical methods to handle missing data (eg, multiple imputation) Missing data: The last observation carry forward method was adopted to fill the validity analysis, that is, the case data that failed to observe the whole treatment process were transferred to the final result of the test with the last observation data. The security evaluation does not evaluate missing data. | ___ Page 13_ _ |
| **Methods: Monitoring** | | |  |
| Data monitoring | 21a | Composition of data monitoring committee (DMC); summary of its role and reporting structure; statement of whether it is independent from the sponsor and competing interests; and reference to where further details about its charter can be found, if not in the protocol. Alternatively, an explanation of why a DMC is not needed DMC consists of the Department of Biomedical Statistics, Southern Medical University, and it mainly responsible for data management and statistical analysis. It is independent from the sponsor and competing interests. | ___ Page 12____ |
|  | 21b | Description of any interim analyses and stopping guidelines, including who will have access to these interim results and make the final decision to terminate the trial No interim analysis was performed in this study. | _____________ |
| Harms | 22 | Plans for collecting, assessing, reporting, and managing solicited and spontaneously reported adverse events and other unintended effects of trial interventions or trial conduct All information regarding adverse events during the study will be recorded in detail, including symptoms, signs, onset time, and severity. Some possible adverse events that may be attributed to RET include reinfection of the root canal, external root resorption, internal root resorption, and discolouration. | _____ Page 11___ |
| Auditing | 23 | Frequency and procedures for auditing trial conduct, if any, and whether the process will be independent from investigators and the sponsor The frequency of audit is once a year. The project organization will review the test process and make comprehensive evaluation. The process will be independent from investigators and the sponsor. | ___ Page 12_ ___ |
| Ethics and dissemination | | |  |
| Research ethics approval | 24 | Plans for seeking research ethics committee/institutional review board (REC/IRB) approval The trial has been approved by the Medical Ethics Committee of Nanfang Hospital, Southern Medical University. This protocol has been reviewed and approved by the sponsor and the ethical committees. Subsequent to initial review and approval, the sponsor and the ethical committees will review the protocol at least annually. The Investigator will make safety and progress reports to the ethical committees at least annually and within three months of study termination or completion at her site. | _____________ |
| Protocol amendments | 25 | Plans for communicating important protocol modifications (eg, changes to eligibility criteria, outcomes, analyses) to relevant parties (eg, investigators, REC/IRBs, trial participants, trial registries, journals, regulators) Any amendments to the protocol will be reviewed and approved by the ethics committee and funding support departments. | ____ Page 13____ |
| Consent or assent | 26a | Who will obtain informed consent or assent from potential trial participants or authorised surrogates, and how (see Item 32) Trained Researchers will introduce the trial to patients. Patients who agree to participate in this study will sign an informed consent form. | _____________ |
|  | 26b | Additional consent provisions for collection and use of participant data and biological specimens in ancillary studies, if applicable Not applicable | _____________ |
| Confidentiality | 27 | How personal information about potential and enrolled participants will be collected, shared, and maintained in order to protect confidentiality before, during, and after the trial All study-related information will be stored in locked file cabinets in the computer securely. All reports, data collection, process, and administrative forms will be identified by a coded ID number. | _____________ |
| Declaration of interests | 28 | Financial and other competing interests for principal investigators for the overall trial and each study site All academic conferences and research activities related to this study will be reimbursed. | _____________ |
| Access to data | 29 | Statement of who will have access to the final trial dataset, and disclosure of contractual agreements that limit such access for investigators The Data Management Coordinating Centre will oversee the intra-study data sharing process, with the involvement of the Data Management Subcommittee. Only the Principal Investigators will be given access to all the data with a special password. Other project investigators will have direct access to their own site’s data sets, and other sites’ data by request. To ensure confidentiality, data dispersed to project team members will be blinded to any identifying participant information. | Page 13-14____ |
| Ancillary and post-trial care | 30 | Provisions, if any, for ancillary and post-trial care, and for compensation to those who suffer harm from trial participation Patients who participated in the study could receive compensation from the study unit, including additional medical care, compensation or damages. | ____ Page 14____ |
| Dissemination policy | 31a | Plans for investigators and sponsor to communicate trial results to participants, healthcare professionals, the public, and other relevant groups (eg, via publication, reporting in results databases, or other data sharing arrangements), including any publication restrictions After the completion of the experiment, the researcher submitted the experimental results to the ethics review, and then published the results data paper. | _____________ |
|  | 31b | Authorship eligibility guidelines and any intended use of professional writers All the researchers involved in the experiment can be the authors of the paper and enjoy the research results of this study. | _____________ |
|  | 31c | Plans, if any, for granting public access to the full protocol, participant-level dataset, and statistical code After 3 years, we will provide complete data sets to the appropriate data files for sharing. | _____________ |
| Appendices |  |  |  |
| Informed consent materials | 32 | Model consent form and other related documentation given to participants and authorised surrogates Appendix: Informed Consent | _____________ |
| Biological specimens | 33 | Plans for collection, laboratory evaluation, and storage of biological specimens for genetic or molecular analysis in the current trial and for future use in ancillary studies, if applicable No plans for collection, laboratory evaluation, and storage of biological specimens for genetic or molecular analysis in the current trial and for future use in ancillary studies. | _____________ |

*It is strongly recommended that this checklist be read in conjunction with the SPIRIT 2013 Explanation & Elaboration for important clarification on the items. Amendments to the protocol should be tracked and dated. The SPIRIT checklist is copyrighted by the SPIRIT Group under the Creative Commons “[Attribution-NonCommercial-NoDerivs 3.0 Unported](http://www.creativecommons.org/licenses/by-nc-nd/3.0/)” license.
